# Evaluation of Upper Body and Lower Limbs Kinematics through an *IMU*-Based Medical System: A Comparative Study with the Optoelectronic System

**DOI:** 10.3390/s23136156

**Published:** 2023-07-05

**Authors:** Serena Cerfoglio, Paolo Capodaglio, Paolo Rossi, Ilaria Conforti, Valentina D’Angeli, Elia Milani, Manuela Galli, Veronica Cimolin

**Affiliations:** 1 Department of Electronics, Information and Bioengineering, Politecnico di Milano, Piazza Leonardo da Vinci 32, 20133 Milano, Italy; manuela.galli@polimi.it (M.G.); veronica.cimolin@polimi.it (V.C.); 2 Orthopaedic Rehabilitation Unit and Research Laboratory in Biomechanics, Rehabilitation and Ergonomics, San Giuseppe Hospital, IRCCS Istituto Auxologico Italiano, Strada Luigi Cadorna 90, 28824 Piancavallo, Italy; p.capodaglio@auxologico.it; 3 Department of Surgical Sciences, Physical Medicine and Rehabilitation, University of Turin, 10126 Turin, Italy; 4 Clinica Hildebrand, Centro di Riabilitazione Brissago, Via Crodolo 18, 6614 Brissago, Switzerland; p.rossi@clinica-hildebrand.ch; 5 Euleria Health Società Benefit Rovereto, 38068 Trento, Italy; ilaria@euleria.it (I.C.); valentina@euleria.it (V.D.); elia@euleria.it (E.M.)

**Keywords:** rehabilitation, tele-rehabilitation, inertial sensors, motion analysis, functional assessment, exercise therapy

## Abstract

In recent years, the use of inertial-based systems has been applied to remote rehabilitation, opening new perspectives for outpatient assessment. In this study, we assessed the accuracy and the concurrent validity of the angular measurements provided by an inertial-based device for rehabilitation with respect to the state-of-the-art system for motion tracking. Data were simultaneously collected with the two systems across a set of exercises for trunk and lower limbs, performed by 21 healthy participants. Additionally, the sensitivity of the inertial measurement unit (*IMU*)-based system to its malpositioning was assessed. Root mean square error (*RMSE*) was used to explore the differences in the outputs of the two systems in terms of range of motion (*ROM*), and their agreement was assessed via Pearson’s correlation coefficient (PCC) and Lin’s concordance correlation coefficient (CCC). The results showed that the *IMU*-based system was able to assess upper-body and lower-limb kinematics with a mean error in general lower than 5° and that its measurements were moderately biased by its mispositioning. Although the system does not seem to be suitable for analysis requiring a high level of detail, the findings of this study support the application of the device in rehabilitation programs in unsupervised settings, providing reliable data to remotely monitor the progress of the rehabilitation pathway and change in patient’s motor function.

## 1. Introduction

Rehabilitation interventions are often extended to home-based settings, and they are crucial to reduce the impact of disability and to optimize the functional recovery of individuals with a broad range of health conditions, including acute or chronic diseases, illnesses, or injuries [1,2]. Rehabilitation usually complements other interventions, such as medical and surgical ones, and it can help to prevent, slow down or manage complications and disabling symptoms associated with many conditions, such as musculoskeletal diseases (e.g., spinal cord injury, fractures, multiple sclerosis) [3,4,5,6]. Motor deficits can introduce various degrees of impairment that need to be addressed separately with specific rehabilitative paths. The ability to accurately measure human movement to assess the functional limitations associated with a pathological state is thus an essential part of clinical evaluation, enabling the determination of the effectiveness of therapeutic interventions [7].

Complex laboratory set-ups based on 3D optoelectronic motion capture (*MoCap*) systems are the gold standard for assessing body kinematics during several motor tasks, providing a complete and accurate description of an individual’s movement and identifying pathological patterns [8]. In order to reconstruct the movement of a bony segment, reflective skin markers are attached over anatomical landmarks, and kinematics are calculated from the position of the markers by fitting the detected marker location to a human body model [8,9,10]. Despite their great accuracy and reliability, such systems are not easily applied as large-scale screening tools because of their high costs and because their use is restricted to a dedicated clinical laboratory set-up [11,12].

In the last decade, the development of new technologies for motion analysis based on wearable inertial sensors has opened new perspectives in everyday clinical practice and in the provision of patient-centric rehabilitative solutions [7,13].

In general, an inertial measurements unit (*IMU*) is a small stand-alone device that integrates different multiaxial sensors (i.e., accelerometer, gyroscope, and magnetometer). The raw data collected by each integrated sensor are complemented via on-board sensor-fusion algorithms based on Kalman filters to achieve a complete description in terms of motion, rotation, and heading with respect to the sensor’s own reference frame. *IMU*s can address different body parts to enable direct determination of kinematics, providing data of angular acceleration, velocity, and spatial orientation [14,15,16,17].

*IMU*-based systems are inexpensive, portable, easy to wear and to set up, and they require neither the use of cameras nor complex laboratory settings, enabling motor assessment in unconstrained environments, such as domestic ones [7,18]. Various systems using body-worn *IMU*s have been used to estimate body kinematics across various motor tasks, starting from standard gait analysis [19,20,21,22] to more complex motor tasks [23], such as jumps [24], squats [25,26], and single-leg squats [27]. Despite generally concurrent validity with traditional motion capture systems [28,29], the validity of *IMU*-based joint kinematics still needs to be further investigated.

A drawback connected to *IMU*-based systems may be related to orientation estimation, which may be prone to drift due to the integration of noisy gyroscope measurements [30]. Although the incorporation of magnetometer data providing a global heading reference can be used to compensate for the error in the transverse plane [16], magnetometers themselves can be susceptible to local disturbance in the magnetic field [16,17,23]. Different approaches have been implemented to limit drift, either correcting magnetic disturbances or omitting magnetometers at all, relying only on acceleration and gyroscope data to estimate joint orientations [31].

The accuracy of *IMU*-based joint kinematics has been explored in several studies. For instance, Seel et al. [22] assessed the performance of two different *IMU*-based systems with respect to a *MoCap* system on transfemoral amputees. With respect to *MoCap*, both inertial systems achieved a root-mean-square deviation lower than 0.6° on the prosthesis side and more than 3° on the contralateral side for the knee joint flexion/extension angle, thus supporting the concurrent validity of *IMU*-based kinematics. However, it should be noted that walking remains a fairly simple task as no large range of motion is achieved and many joints are solicited mostly across a preferential degree of freedom as in the case of knee flexion [23].

Functional assessment movements used in rehabilitation and in sports medicine differ from common gait as they incorporate almost no global translation, usually demanding higher *ROM* and higher global accelerations [31]. In this context, *IMU*s seem to be a suitable tool in the detection of relevant parameters in in-field-based diagnostics, for instance in jumping skills, even though the validity of *IMU*s to assess slow and dynamic functional movements needs to be further explored. In fact, research comparing *IMU*s and *MoCap* reported that comparability in joint angles may be task-related, thus limiting the generalization of the results [23].

*IMU*-based systems have been successfully used in the rehabilitation field for inpatient assessment [32,33,34,35] and to support the administration of remote home-based rehabilitation programs [36,37,38]. The technologies designed for tele-rehabilitation usually integrate body-worn inertial sensors into exergame- and biofeedback-based systems [39,40,41], allowing for the provision of real-time visual or audio feedback to the user regarding their movement and its correctness [18,42].

In unsupervised rehabilitation programs in home settings, a risk of bias on intervention effectiveness is usually related to incorrect or poor performance of assigned motor tasks [18], as well as to the tendency to over-report the number of times patients carried out home-based exercises [36]. Wearable technologies can enable the automated, unsupervised, and objective evaluation of home-based exercise programs, together with patients’ compliance with the prescribed treatment plan [36]. Additionally, the biofeedback encourages the patient to pay more attention to the execution of exercises [42], also through a gamification mechanism based on scores, badges, and prizes that may increase their motivation and the engagement in the program [43,44,45].

Together with appropriate back-end infrastructures, the integration of such technologies enables the development of innovative solutions for real-time monitoring and for continuous remote supervision by clinicians [13]. Although the use of various systems using body-worn sensors to objectively measure body kinematics has been validated with respect to the gold standard, their accuracy and reliability need to be further evaluated for successful adoption in the rehabilitation field to define best practices and standardized protocols [13].

The aim of this study was two-fold. First, the accuracy of kinematic measurements, in terms of body segment orientation, provided by the inertial system, was assessed during the execution of a set of motor tasks for trunk and lower limbs using an optoelectronic-based system as the gold standard. Second, the sensitivity in tracking kinematic parameters was assessed with respect to *IMU*’s malposition, as incorrect positioning could affect the performance of the system [42].

## 2. Materials and Methods

The study took place from November 2022 to February 2023, and it involved the simultaneous data collection with an *IMU*-based system (Euleria home, Euleria Health, Rovereto, Italy) and an optoelectronic marker-based motion capture system (SMART DX 400 system, BTS Bioengineering SPA, Milan, Italy).

Twenty-one healthy subjects (M: 10/F: 11; age: 23.5 ± 1.3 years; height 175.3 ± 8.7 cm; weight 68.5 ± 11.6 kg; BMI 22.1 ± 2.1 kg/m^2^) were recruited on a voluntary basis.

The inclusion criteria were healthy weight (BMI: 18.5–24.9 kg/m^2^) and the absence of participant’s self-reported neurological or musculoskeletal conditions. The study, which was carried out in accordance with the ethical standards of the institution and the 1964 Helsinki declaration and its latest amendments, was approved by the ethical committees of Politecnico di Milano (22/2021, 14 June 2021). Written informed consent was signed by all participants.

### 2.1. Experimental Set-Up

#### 2.1.1. *MoCap* and Marker Set

The *MoCap* system used for optical data collection was the 8-cameras (sampling frequency: 100 Hz) SMART DX 400 system (BTSBioengineering SPA, Milan, Italy).

Participants’ anthropometric data, i.e., (i) height, (ii) weight, (iii) distance between the femoral condyles or diameter of the knee, (iv) distance between the malleoli or diameter of the ankle, (v) distance between the anterior iliac spines, and (vi) thickness of the pelvis, were measured. A set of 22 passive markers was placed over the anatomical landmarks of each subject according to the marker set-up proposed by Davis et al. [46]. The location of the landmarks was determined manually by palpation, identifying regions of reduced tissue thickness interposed between bone and skin. During the test campaign, an additional optical marker was placed on the *IMU* to use its signal as a reference for data synchronization.

#### 2.1.2. *IMU*-Based System

The Euleria home (Euleria Health, Rovereto (TN), Italy) is a medical device for home rehabilitation. It is composed of an *IMU* connected wirelessly with technology BLE to a tablet with a dedicated application installed, guiding the patient through the execution of an exercise-therapy program ad hoc configured by the clinician. The patient is instructed to wear the *IMU* on a specific body segment through a dedicated elastic band. The elastic band is embedded with a magnet, limiting its movements during the execution of the exercises. These are performed while an audio–video guide guides the user to perform the right movements. Moreover, real-time feedback is provided by the system in order to inform the patient on the quality of the movement with respect to the target, thanks to the *IMU*’s output and the processed kinematic parameters.

The *IMU* (*IMU*s, Xsens DOT, Movella Technologies—NL, size: 36.3 × 30.4 × 10.8 mm, weight: 11.2 g) integrates different tri-axial sensors, i.e., accelerometer, gyroscope, and magnetometer, allowing for a complete description of motion in its own three-dimensional local coordinate system [14,22]. The sampling rate was set at 30 Hz. The orientation gathered through the *IMU* allows the algorithm to compute the Eulerian angles as roll (rotation around the body), pitch (vertical incline), and yaw (heading direction), expressed in degrees, with a measurement error below 5% as reported in the data sheet. Considering the *IMU*’s size and the elastic bands, it can be easily worn on body segments following the guidelines of the app before starting the task. Once the *IMU* is worn, the user just needs to start the app from the tablet and follow the audio–video guide and biofeedback trace on the tablet screen to perform the tasks.

### 2.2. Study Design

**Figure 1:**
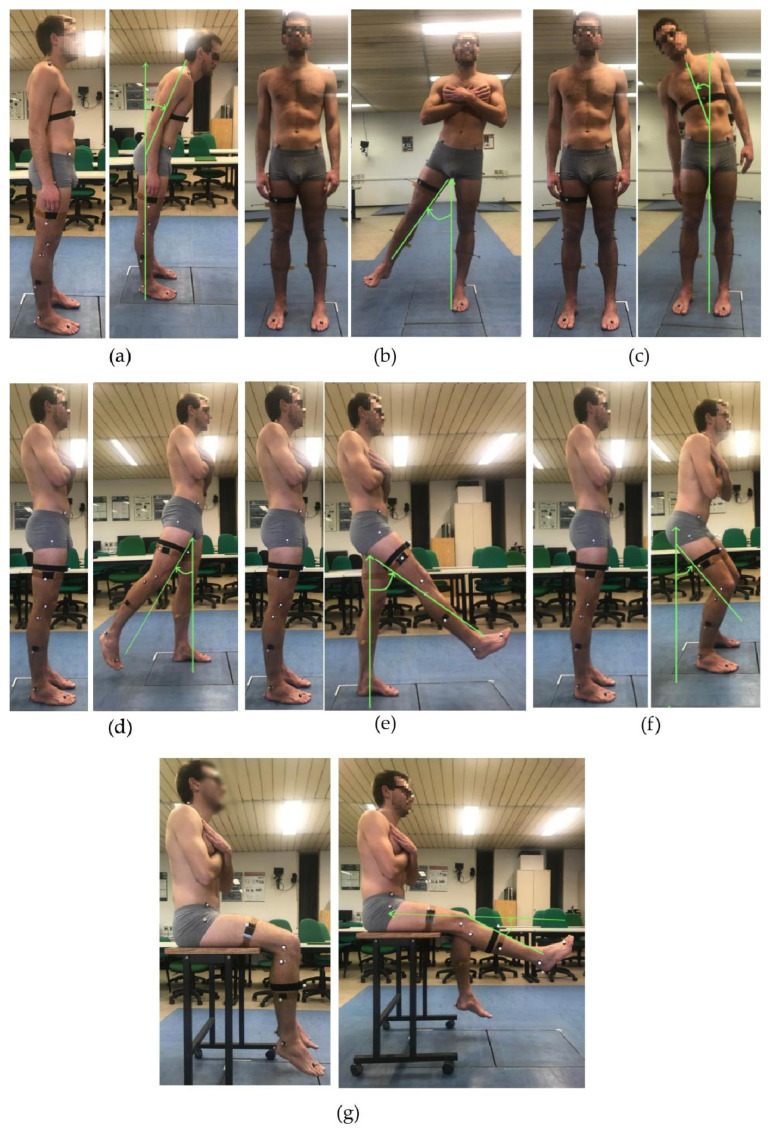
A volunteer equipped with the *IMU* and the marker set during data collection of the experimental protocol: anterior trunk flexion (**a**); trunk bending (**b**); hip abduction (**c**); hip extension (**d**); hip flexion (**e**); semi-squat (**f**); and knee extension from a seated position (**g**).

A set of seven motor tasks for trunk and lower limbs was selected from the device’s exercise library via the cloud-based web management system and performed by each participant simultaneously equipped with the *IMU* and the marker set (Figure 1). In particular, the following motor gestures were performed:

- Anterior trunk flexion (Figure 1a);
- Trunk bending towards right/left (Figure 1b);
- Right/left hip abduction (Figure 1c);
- Right/left hip extension (Figure 1d);
- Right/left hip flexion (Figure 1e);
- semi-squat (Figure 1f);
- Right/left knee extension from a seated position (Figure 1g).

**Figure 2:**
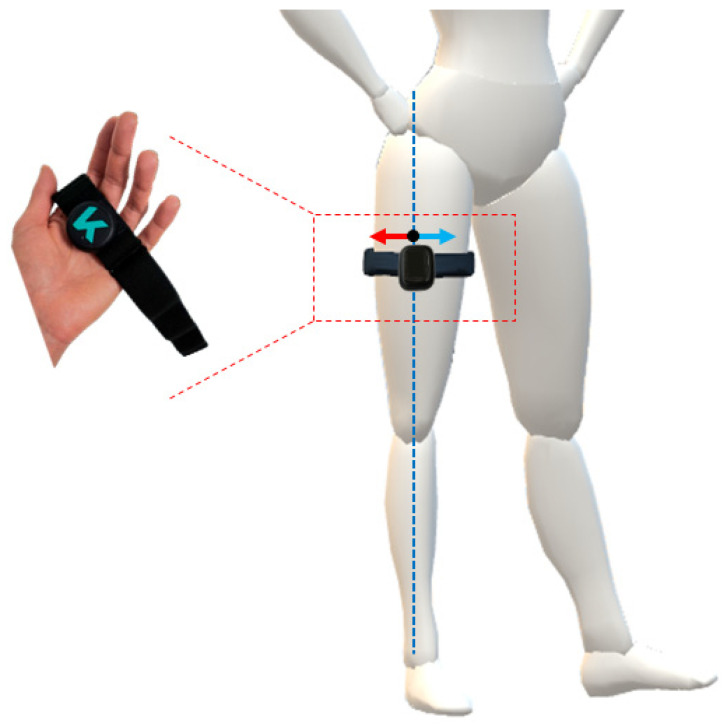
Visual representation of the *IMU* worn on the thigh via elastic band. The correct placement of the *IMU* is along the dotted blue line, whilst the two incorrect configurations are achieved by moving the *IMU* medially (blue arrow) and laterally (red arrow) along the diameter of the thigh.

To evaluate the sensitivity of the outcome measurements of the *IMU*-based system, considering the two conditions of *IMU*’s placement, i.e., (i) correct and (ii) incorrect, bilateral hip flexion and extension tasks were performed with incorrect placement. In particular, the incorrect condition has two sub-conditions; thus, the *IMU* can be positioned medially or laterally with respect to its neutral, or correct, position along the thigh as displayed in the device (Figure 2). The positions with the displaced *IMU* were calculated as 10% of the subject’s thigh diameter to move the sensor laterally or medially along the thigh with respect to the correct position.

The number of repetitions was set to 10 and all the motor tasks were performed according to the audio and visual feedback provided by the *IMU*-based device. As for the synchronization between the *IMU*-based device and the gold standard, a motor artifact was used as a trigger. According to a previously reported method, the artifact was created by asking the subject to quickly step on the floor in order to obtain a few spikes visible from both systems, which was useful for signal post-processing [47].

### 2.3. Data Analysis and Processing

The angles measured by the *IMU* were automatically computed throughout the execution of each task and stored in the cloud associated with the mobile app in a .csv file, which also contained the raw acceleration and raw gyroscope data. The body segment angles were computed extracting the Euler angle from quaternions output with the ZYX-axis rotation sequence in terms of flexion/extension, internal/external rotation, and adduction/abduction, respectively. In particular, they were expressed as absolute body segment angles in *IMU*’s coordinate system.

Raw optical data were processed with SmartTracker and SmartClinic (BTS Bioengineering, Milan, Italy). Data were tracked and interpolated to obtain the 3D reconstruction of the coordinates of each marker. According to the algorithm embedded in the software, instantaneous orientation of an orthogonal marked-based, embedded coordinate system was determined for the trunk and pelvis, as well as for the thigh, shank, and foot segments. The embedded coordinate systems of the lower body segment were then realigned with the instantaneous, joint-center-based, body-fixed coordinate system, as described by Davis et al. [46]. Finally, 3D segment rotation angles were computed from the embedded coordinate system information, and rotation matrices defined for each joint were used to compute body segment angles, relying on Euler angles [48]. In particular, a YXZ-axis rotation sequence in terms of flexion/extension, adduction/abduction, and internal/external rotation, respectively, was used.

According to Davis’ protocol, hip, knee, and ankle joint rotation angles are computed as relative angles (for example, hip angles correspond to the orientation of the thigh with respect to the pelvis), while trunk and the pelvic angles are absolute angles computed with respect to the inertially fixed laboratory reference frame [46]. Since the *IMU*-based system computes thigh angles as absolute thigh segment angles, optical-based hip kinematics were computed as absolute angles to adopt the same convention as the *IMU* and to compare the output of the two systems.

### 2.4. Data Synchronization and ROM Computation

The comparison of the outcomes of the two systems benefited from a synchronization method that used movement artifacts, as described above. The time synchronization of the kinematic measurements was performed according to Cerfoglio et al. [47]. Firstly, the raw trajectory of the marker placed on the *IMU* along the y-axis of the global reference system of the laboratory, acquired at 100 Hz, was down-sampled to 30 Hz, i.e., the *IMU*’s sample frequency. The raw acceleration data along the x-axis of its own reference system was filtered using a standard third-order low-pass Butterworth filter with a 5 Hz cut frequency. The parts of the signals containing the spikes, representative of the movement artifacts, to be matched were isolated, and cross-correlation between them was computed. The resultant delay was used to align the angular measurements of both systems.

The offset between the measurements gathered through the reference system and the *IMU*-based system was removed, and each repetition of each task was isolated by using a custom-made Matlab script using the position of local minima to locate each repetition in the signal. Repetitions were then resampled to standardize the number of frames.

Considering the orientation of the body segment engaged in the task, the range of motion (*ROM*) was computed as the difference between the final position during the working phase and the mean value of the angle relative to the working phase. Specifically, in the *IMU*-based system used in this study, the execution of the task concerns the rest and working phase to let the patient control their motion, handling and stabilizing the *ROM* thanks to the biofeedback and the visual guide throughout the session. Therefore, the amplitude of each repetition was normalized with respect to its maximum value to isolate the working phase and compute its mean value. Thus, the algorithm records how well the patient maintains their maximum *ROM* during the exercise, so to calculate the *ROM* just as the difference between the maximum and minimum value of the measured angle is not effective.

Since no significant difference (*p* > 0.05) was found in the motion pattern between right and left side in the tasks performed bilaterally, data were pooled and the *ROM*s were computed over the total number of repetitions (i.e., 20 repetitions, 10 per side) instead of considering them separately for each side.

Regarding the body segment angle measured through the *IMU* placed in the incorrect position, the *ROM* was calculated with the same method and the comparison was made considering the *ROM* obtained during the exercise executed with the sensor placed in the incorrect position and the *ROM* of the correct position.

### 2.5. Statistical Analysis

Statistical analysis was performed with Matlab, Minitab Statistical Software (2023 Minitab, LLC, State College, PA, USA), and MedCalc (2023 MedCalc Software Ltd., Ostend, Belgium).

Data were checked for normality via the Anderson–Darling test. As data were normally distributed, variables were computed in terms of mean and standard deviation. The accuracy of the parameters evaluated through the *IMU*-based device was computed according to the following equation (Equation (1)):

(1) $$Accuracy=1-\frac{\left|ROM_{IMU}-ROM_{MoCap}\right|}{ROM_{MoCap}}\times 100$$

Pearson’s correlation coefficient (PCC) was calculated to describe the agreement between the kinematic measurements obtained from the two systems. For each task, PCC was computed considering the mean *ROM* values of each participant. PCC represents the degree to which a straight line fits the data. To assess agreement between two systems, all matched data points should fall on the equality line. However, PCC searches for the best fitting line for the data, which is not per se the equality line, potentially leading to a systematic bias [49,50]. For this reason, an additional correlation coefficient (Lin’s concordance correlation coefficient CCC) was calculated. CCC expresses the concordance between bivariate pairs of observations between new technology (i.e., *IMU*) and gold standard technology (i.e., *MoCap*), ranging from −1 to 1, with perfect agreement at 1 [51].

Bland–Altman plots were also generated and used to support and describe the agreement between the two systems. Bland–Altman analysis is also useful to highlight if a method overestimates high values and underestimates low values.

Additionally, the root mean square error (*RMSE*) was computed according to the following equation (Equation (2)):

(2) $$RMSE=\sqrt{\overset{n}{\underset{i=1}{\sum }}\frac{{\left(\hat{y_{i}}-y_{i}\right)}^{2}}{n}}=\sqrt{\overset{n}{\underset{i=1}{\sum }}\frac{e_{i}^{2}}{n}}$$

where *ŷ_i_*…*ŷ_n_* are the *ROM*s computed from the *MoCap*, *y_i_*…*y_n_* are the *ROM*s computed from the *IMU* (thus, *e_i_*…*e_n_* are the errors), and *n* is the number of observations, i.e., the number of participants.

Finally, the standard error of measurement (*SEM*) was computed for each task according to Equation (3):

(3) $$SEM=SD\;\sqrt{1-R\;}=SD\sqrt{1-\frac{\sigma_{MoCap}^{2}}{\sigma_{IMU}^{2}}}$$

where *SD* is the standard deviation associated with the measurements of both systems, and R is the reliability coefficient, computed as the ratio between the variance of *MoCap* and *IMU*’s measurements on the overall sessions performed by the participants. *SEM* can be directly related to both system reliability and to the clinical significance of a measurement. With respect to the latter, one *SEM* was proposed as a reference threshold to identify the minimal clinically important difference (MCID), defined as the minimal change that can be detected and that is relevant to the patient [52]. When comparing the outputs of two different systems, the differences between the gold standard and a concurrent system should not exceed the MCID to make sure the that the alternative system is able to detect meaningful changes in the outcome measurements, reflecting the progress of rehabilitation treatment, and therefore important in interpreting the clinical relevance of the observed change [53].

With respect to sensitivity analysis of the kinematic parameters obtained in the *IMU*’s misplacement conditions, the *RMSE* was computed according to Equation (2), where *ŷ_1_*…*ŷ_n_* are the *ROM*s of the measurements obtained from misplaced *IMU* and *y_1_*…*y_n_* are the *ROM*s of the measurement obtained from the *IMU* in its correct position. The *RMSE* was computed for each malposition case, i.e., medial or lateral displacement.

## 3. Results

### 3.1. MoCap and IMU Data Comparison

Table 1 reports the mean and the standard deviation values, together with the accuracy and the *RMSE* for the joint angles in terms of *ROM* and the MCID associated with each motor task. The *ROM* values appear to be coherent between the two systems, although the difference varies across motor tasks. For hip extension and anterior trunk flexion, the corresponding *ROM* values report a discrepancy of up to 10 degrees, higher than in the other tasks, as confirmed by the corresponding values of accuracy and *RMSE*.

Regarding the MCID, the average difference between the two systems is below the threshold (one *SEM*), just for semi-squat and hip flexion tasks, whilst it variably exceeds the MCID in the remaining tasks.

**Figure 3:**
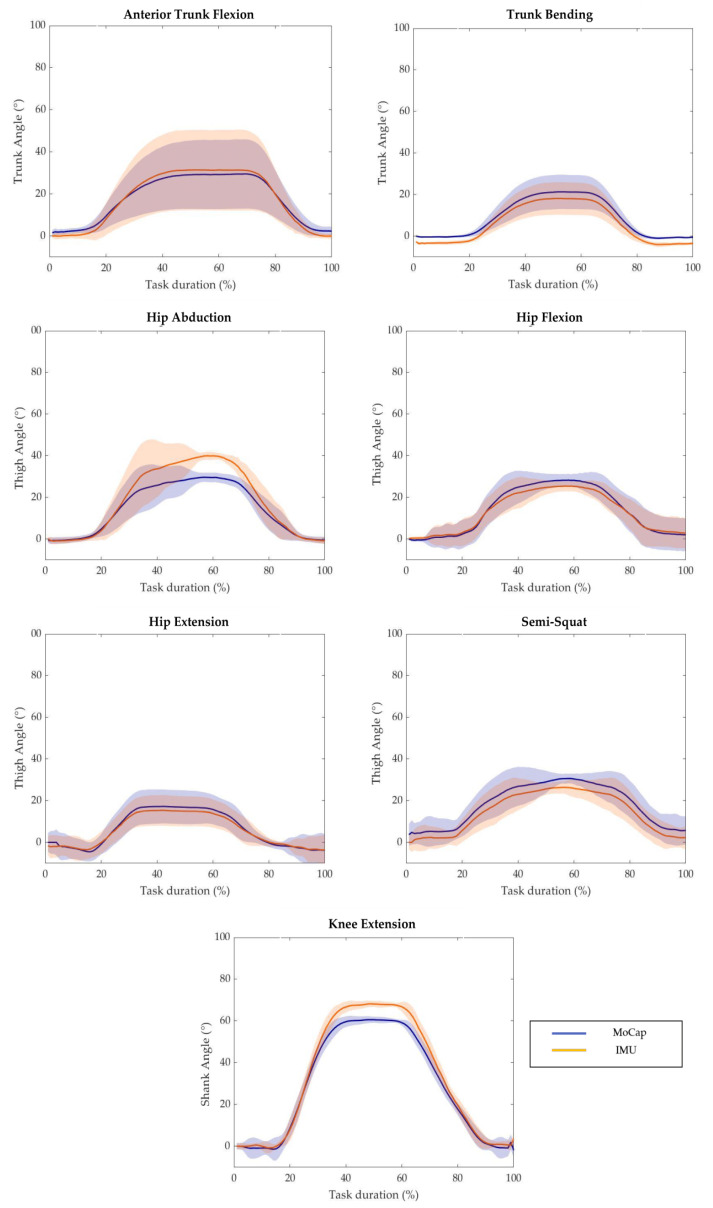
Average value of *MoCap* system output (blue solid line) and *IMU*-based device output (orange solid line) with respect to the task duration of a representative subject performing each task of the experimental protocol. The shaded areas represent the standard deviation.

The mean values of the body segment angles measured through the *MoCap* and *IMU*-based system across the different motor tasks in a representative subject is displayed in Figure 3. The curves yield qualitative assessment of how the output of two systems follow the same pattern. In fact, slight-to-moderate differences can be observed during the working phase for almost all tasks of the experimental protocol.

### 3.2. Agreement Analysis between MoCap and IMU Measurements

Concerning the agreement analysis between the sets of data retrieved from the two systems, the corresponding values of PCC and CCC are reported in Table 2. As it can be observed, the PCC values between the measurements gathered through the two systems are all statistically significant (*p* < 0.05) and higher than 0.75, with the exception of anterior trunk flexion (PCC = 0.68, *p* < 0.05), highlighting the validity of the *IMU*-based system to measure the body segment angles during the selected motor tasks. The same considerations are confirmed by the corresponding CCC values, where a value higher than 0.7 indicates strong agreement between the measurements.

**Figure 4:**
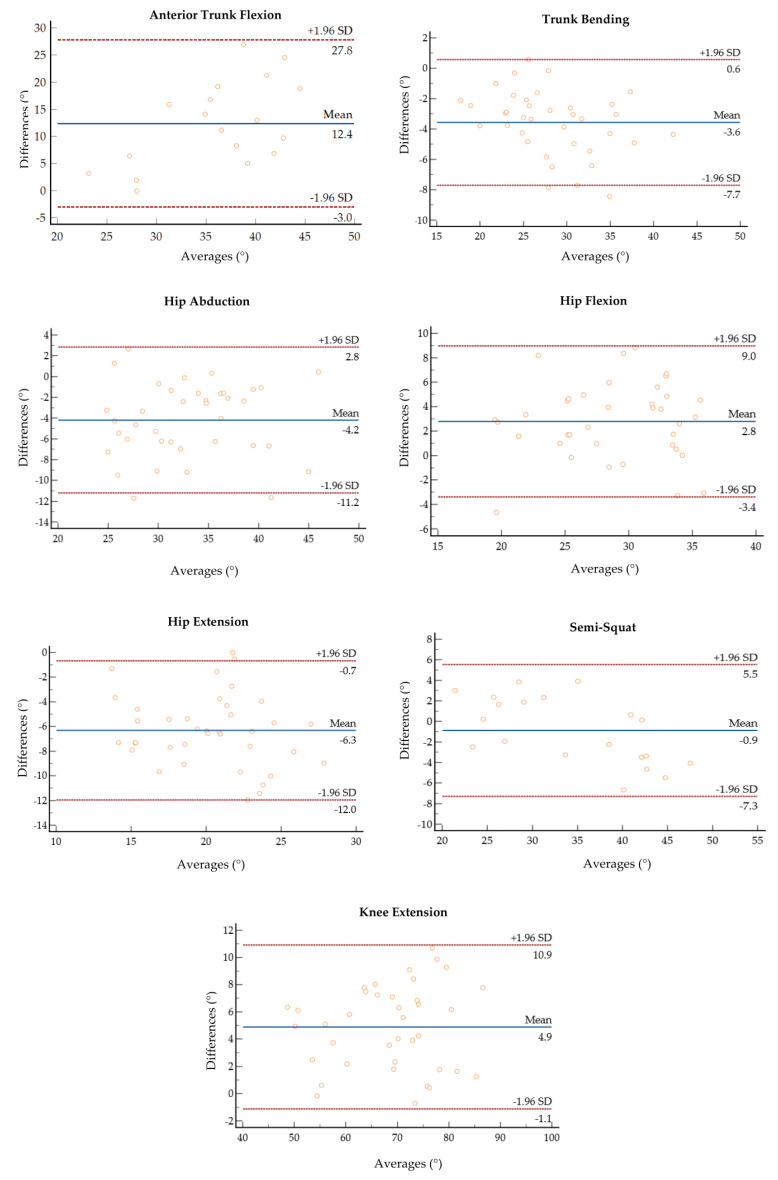
Bland–Altman plots of the average value of *ROM* retrieved from the *MoCap* and the *IMU* plotted against the difference between the two systems for each motor task.

Figure 4 reports the correlation and the Bland–Altman plots for all the estimated body segment angles. Bland–Altman analysis is a graphical method that enables the evaluation of the agreement between two different measurements and the verification of where 95% of the difference falls [54]. The horizontal lines indicate the mean difference and the LoA (limit of agreement), defined as the mean difference ±1.96 * standard deviation. The differences between the two paired values are displayed as y-values, whilst their mean is reported as x-values. In the current analysis, 95% of the differences fall inside the LoA, indicating a globally good association between the gold standard and the *IMU*-based device. The Bland–Altman outputs are coherent with the values of accuracy between the two systems reported in Table 1.

### 3.3. Sensitivity Analysis of IMU’s Measurements to Malposition

With respect to the sensitivity analysis of *IMU* measurements to lateral and medial *IMU* displacement, the mean and the standard deviation for hip flexion and extension in terms or *ROM* are reported in Table 3, together with the values computed with the *IMU* in a correct position and the *RMSE* computed as described in Section 2.5. As can be observed, the *ROM* values for lateral and medial misplacement show a difference to the order of 1 to 7 degrees with respect to the reference values of the correct configuration of the *IMU*.

## 4. Discussion

The Euleria home is an *IMU*-based medical device for home therapy. Following a game-based approach, biofeedback technology, and integration with a body-worn *IMU*, it guides the user in the execution of rehabilitative motor tasks.

The gold standard to quantitatively assess the effects of rehabilitative treatment on motor performance is represented by complex laboratory set-ups based on optoelectronic motion capture systems. In the last decade, the development of new technologies based on wearable *IMU*s has opened new perspectives in both functional inpatient and outpatient assessment. However, prior to their use in everyday clinical practice, new systems to track human movement should be validated against the gold standard. In the present study, the concurrent validity and the accuracy of *IMU*-based joint kinematics was assessed across the execution of a set of motor tasks for trunk and lower limbs with respect to a gold standard system for motion analysis.

The aggregated results highlight and support the dependability of the *IMU*-based body segment angle measurements from either a qualitative or quantitative point of view, although the concordance between the systems varies across the selected motor tasks. From the results, it can be observed that the overall trend in measured angular traces retrieved from the *IMU* are coherent with those obtained from the *MoCap* for each motor task, with a slight-to-moderate difference at the peak of the working phase.

With respect to the motor tasks involving trunk bending and anterior flexion, differences in *ROM* and accuracy values can be found in the estimation of the angles between the two motor tasks. In fact, whilst the systems seem to compare well in the computation of the lateral bending angle and the achieved *ROM* and *RMSE* are in line with those reported in the literature [42,55], the *IMU* appears to overestimate about 10° the anterior flexion angle, with a 66% accuracy value. Although such a value seems to be in contrast with previous studies reporting a mean difference lower than 3° between the measurements of an inertial-based system and the gold standard, it should be noticed that other studies used complex systems based on multiple *IMU*s instead of a single *IMU* [42,56]. In fact, data fusion of multiple *IMU*s usually yields a more accurate estimation of the kinematics of the specified segment with respect to a single *IMU* [22]. From this perspective, the *IMU*-based system tested in the present study reached interesting results considering the set-up differences.

The reported difference between the two systems in the estimation of the trunk anterior flexion angle can be also due to the adopted protocol for marker placement and trunk segment definition. According to Davis’ protocol [46], the trunk is defined as a single segment by the markers placed on the seventh cervical vertebrae and the shoulders, and the markers on the pelvis and the anterior flexion angle are computed accordingly. As the body-worn *IMU* was placed on the thorax, it can be hypothesized that a single *IMU* may not be able to capture the full movement of a wide body segment as the trunk defined according to Davis et al. [46]. Instead, the application of other markers, which allow for the division of the trunk in different areas [57], could better estimate the considered angle. Further investigations are required on this specific aspect, e.g., compensating the difference by introducing a correction factor improving the body segment orientation estimation.

Concerning the motor tasks for lower limbs, the mean difference between the systems in the estimation of thigh and shank body segment angles during knee extension, hip abduction, and flexion/extension tasks is in line with the literature [42,56]. As for thigh body segment angles, the two systems seem to compare very well in the estimation of the body segment angle corresponding to hip flexion, whilst the difference between the two systems is more of a marker for hip abduction and extension tasks. The difference in the accuracy of the computation across the hip tasks can be both due to the placement of the *IMU* itself and to participants’ own motor patterns.

Hip joint mobility depends on different factors, and it can vary also among healthy individuals [58,59]. In case of limitation of hip joint *ROM*, the individual may adopt compensatory strategies, such as hip external rotation, to achieve full extension. These compensatory strategies could be appreciated in more than one anatomical plane; thus, a single *IMU* is not effective to assess joint changes. Nevertheless, the measured orientation or body segment angle may not be effective in discriminating the pattern change, especially for joints with a high range of motion. Assessing joint mobility is necessary to increase the number of sensors that are computing relative angles, but this means losing the advantages relative to the adoption of one *IMU* to monitor and assess the quality of movements.

Concerning the MCID and thus the ability of the *IMU*-based system to detect meaningful changes in a patient’s condition, the results are in line with previously discussed aspects. Although there is generally good agreement from either a qualitative or quantitative point of view, the *IMU*-based system does not appear to be suitable for analysis requiring a high level of detail since it does not seem to be able to detect change in the *ROM* lower than 2° across the explored tasks. However, further investigations on these aspects are needed. In the literature, several methods have been proposed to compute MCID, but there is no clear consensus regarding which methods are the most suitable [53]. In addition, since this work focused on healthy subjects, it could be interesting to evaluate the performance of patients following a tele-rehabilitation program with the proposed *IMU*-based system and compare their pre/post-sessions to check if relevant changes in their motion pattern are detected by both the gold standard and the *IMU*-based system.

However, despite the discussed limitations and differences, the results indicate generally good agreement, accuracy, and correlation between the kinematics estimated by the two systems across the tasks of the experimental protocol. The *IMU*-based system generally allows for an adequate estimation of trunk and lower-limb kinematics, as demonstrated through the comparison with the gold standard. It is important to note the *IMU*-based system was not designed as a replacement for the *MoCap* system, but as an intermediate tool for motion analysis in those environments where gold standard systems are impractical, such as the home and ecological settings in real-life contexts [54]. However, the interesting results of this study highlight the potential design of an *IMU*-based system that is easily integrated in home environment settings for rehabilitation demands for both patients and clinicians.

The *IMU*-based system tested in this study was designed as a self-practice rehabilitation tool and it was not intended for specific diagnostic purposes, but it may provide reliable information on joint kinematics, integrating clinical evaluation in different rehabilitation settings. In tele-rehabilitation contexts, a portable system providing reliable kinematic estimation allows the professional to monitor and keep track of the progress of the rehabilitation pathway and changes in patients’ motor function in unsupervised settings and to tune and modulate the rehabilitation program accordingly. Even though the reported differences between the two systems in terms of accuracy may affect finer analysis and diagnostic purposes, they cannot be determinant in this context.

Considering the second aim of the study, the sensitivity of the device in tracking kinematics through the body segment angle was assessed with respect to either a medial or lateral displacement of the *IMU* during hip flexion and extension tasks.

The *IMU*’s angular measurements are slightly to moderately biased by its malposition. With respect to hip flexion, the *ROM*s corresponding to medial and lateral displacement display a difference of less than −3° with respect to the *ROM* computed with the *IMU* in the correct place. In hip extension, the discrepancy between the *ROM* values rises up to +6° degrees when the *IMU* is laterally displaced. Such results are in line with those of a previous study assessing *IMU* sensitivity to mispositioning [42]. Although just a couple of erroneous configurations were explored, it is reasonable to think that for larger displacements, the system would not be able to detect movement along a precise direction and would display an error message to warn the user. However, it is important to underline that the sensitivity of the *IMU*-based system to location errors in tracking movement could be considered tolerable with a self-worn system used for a domestic rehabilitation program.

Rehabilitation settings are generally less demanding in terms of required accuracy, especially in domestic environments. However, the possibility to track meaningful kinematic parameters that are of the same order of those retrieved from a gold standard system with a portable, automated, affordable, and user-friendly technological solution opens new perspectives in outpatient assessment [54]. In outpatient settings, the use of the proposed *IMU*-based system may also be effective in improving patient’s health and family care, either by reducing the need for hospital evaluations or by guaranteeing continuous assistance and provision of patient-centric rehabilitative solutions.

## 5. Conclusions

The present study aimed to investigate the validity and the accuracy of an *IMU*-based device, designed for rehabilitation, with respect to a gold standard system, as well as its sensitivity to the *IMU*’s misplacement, to compute the body segment’s angle measurements. The overall results confirmed that the *IMU*-based system was able to track body segment’s angles accurately with a mean error generally lower than 5° in terms of *ROM*, and it is slightly affected by errors in positioning the *IMU* itself. The system seems to be suitable to be applied in rehabilitation programs in unsupervised settings, such as domestic ones.

In this study, the participants were all young and healthy and easily performed the selected tasks. In the case of individuals with disabilities or compromised motor function, their motion patterns may significantly deviate from the healthy ones. In order to better generalize the results of this study, it could be interesting to evaluate the performance of the *IMU*-based device in the detection of aberrant motion patterns and to expand its validation to other tasks, also involving upper limbs.

## Figures and Tables

**Table 1:** Mean and standard deviation values, average difference, accuracy, *RMSE*, and *SEM* (i.e., MCID) for the body segment angle estimated with the two systems during each motor task. ^†^ |*MoCap* − *IMU*| > MCID.

Motor Task	*ROM* (°)			Accuracy (%)	*RMSE* (°)	MCID = 1 *SEM* (°)
	*IMU*	*MoCap*	|*MoCap* − *IMU*|	*IMU* vs. *MoCap*	*IMU* vs. *MoCap*	
semi-squat	33.92 (7.34)	34.80 (9.33)	0.88	97.5%	3.71	5
Hip abduction	30.93 (6.09)	35.12 (5.90)	4.19 ^†^	88.1%	6.38	1.74
Hip flexion	30.28 (5.24)	31.32 (5.17)	1.04	96.7%	3.68	1.85
Hip extension	17.08 (3.75)	23.40 (4.26)	6.32 ^†^	73%	8.47	3.78
Knee extension	71.05 (10.28)	66.15 (9.95)	4.9 ^†^	92.6%	5.90	1.57
Anterior trunk flexion	40.15 (8.36)	30.15 (5.50)	10 ^†^	66.8%	14.55	8.07
Trunk bending	26.53 (5.33)	30.10 (6.06)	3.57 ^†^	88.1%	4.49	3.24

**Table 2:** Correlation values of body segment angles estimated through the *IMU*-based system and the *MoCap*: results for Pearson’s correlation (PCC) and concordance correlation coefficient (CCC). * = *p* < 0.05.

Motor Task	Pearson Correlation Coefficient (PCC)	Concordance Correlation Coefficient (CCC)
semi-squat (hip flexion)	0.95 *	0.94 *
Hip abduction	0.82 *	0.76 *
Hip flexion	0.86 *	0.90 *
Hip extension	0.75 *	0.43
Knee extension	0.95 *	0.92 *
Anterior trunk flexion	0.68	0.52
Trunk bending	0.95 *	0.83 *

**Table 3:** Mean and standard deviation values and *RMSE* for the *IMU*-based measurements for hip flexion and extension tasks.

	*ROM* (°)			*RMSE* (°)	
	*IMU*	*IMU*_MEDIAL_	*IMU*_LATERAL_	*IMU* vs. *IMU*_MEDIAL_	*IMU* vs. *IMU*_LATERAL_
Hip flexion	30.28 (5.24)	28.90 (4.88)	27.70 (5.79)	5.12	5.13
Hip extension	17.08 (3.75)	19.98 (3.36)	11.90 (3.61)	4.86	7.09

## Data Availability

Data are available on request due to restrictions, e.g., privacy or ethical.
